# Surgical site infections in sub-Saharan Africa: epidemiology, risk factors, and prevention strategies

**DOI:** 10.1097/MS9.0000000000003188

**Published:** 2025-03-20

**Authors:** Hamza Nakhleh, Boluwatife Samuel Fatokun, Hamiidah Nakyanzi, Sarah Mshaymesh, Jack Wellington, Olivier Uwishema

**Affiliations:** a Department of Research and Education, Oli Health Magazine Organization, Kigali, Rwanda; b The National Center for Diabetes, Endocrinology and Genetics, University of Jordan Amman, Jordan; c School of Medicine, The University of Jordan, Amman, Jordan; d Department of Medical Laboratory Science, Federal Teaching Hospital, Ido, Ekiti, Nigeria; e Faculty of Basic Medical Sciences, Kwara State University, Malete, Nigeria; f Faculty of Nursing and Midwifery, Lira University, Lira, Uganda; g Clinton Global Initiative University, New York, USA; h Faculty of Sciences, Haigazian University, Beirut, Lebanon; i Leeds Teaching Hospitals NHS Foundation Trust, Leeds, United Kingdom

**Keywords:** perspectives, prevalence, preventive measures, solutions, sub-Saharan Africa, surgical site infections

## Abstract

**Introduction:**

The healthcare systems in sub-Saharan Africa (SSA) face many challenges, with surgical site infection (SSI) considered a major health issue concerning its communities. The higher incidence of SSI in comparison to international standards highlights the issue further and advances efforts to develop all-encompassing remedies. With a frequency of up to 41.9% in Tanzania compared to 2.5% in Europe, prompt solutions are warranted. This issue has developed as a result of a dearth of resources, inadequate healthcare infrastructure, inconsistent application of the recommendations, and ineffective data gathering methodologies.

**Approach:**

A comprehensive search of the literature pertaining to the epidemiology, associated risk factors, and challenges attributed to the control of SSIs in SSA was performed employing electronic databases like that of PubMed/MEDLINE and Google Scholar. Published articles discussing these topics were reviewed and evaluated for their contribution to the topic of SSI in SSA then the relevant data were referenced.

**Conclusion:**

There is a significant disparity between SSA and the industrialized world in terms of the occurrence of SSIs. The lack of resources, comprising insufficient clinical infrastructure and financial constraints, is a barrier to the implementation of SSI prevention strategies. Furthermore, it was observed that a large proportion of the medical workforce in nations belonging to SSA disregarded sterilization protocols. In addition, inadequate data collation causes the field to provide inadequate feedback, which raises the risk of SSIs. However, there are approaches that are thought to successfully lower the risk of SSIs. It is essential to utilize gold standard sterilizing procedures. These include pressurized wound irrigation, antimicrobial-coated sutures, perioperative blood glucose regulation, and the administration of chlorhexidine gluconate combined with alcohol-based skin preparation. Further, obtaining external means of funding from global organizations may be considered as a solution to issues regarding resource deprivation.

## Introduction

Hospital acquired infections (HAIs), sometimes referred to as nosocomial infections, include surgical site infections (SSIs) that develop in the vicinity of surgical wounds. The usual window for detecting SSIs is 30 days following surgical intervention; if an implant is left in situ, this window is prolonged to 1 year.^[^1^]^

**HIGHLIGHTS**

- The incidence of surgical site infections (SSIs) in sub-Saharan Africa (SSA) is significantly higher compared to international standards.
- Inadequate data collection limits accurate assessment and feedback, impeding the development of impactful strategies to control and prevent SSIs in SSA.
- The study identifies a lack of resources, insufficient clinical infrastructure, and financial constraints as major obstacles to prevent and manage SSI in SSA effectively.

They may significantly raise morbidity, prolong hospital admissions, and inflict postoperative sequalae. Moreover, SSIs present a serious risk to patient outcomes, healthcare expenditure, and the quality of care provided by medical personnel^[^2^]^. Twenty percent of healthcare related morbidities increase the risk of mortality 2–11 times, according to the Centre for Disease Control and Prevention^[^3^]^.

Morbidity rises significantly in cases of SSI. This includes delayed wound healing, cellulitis, abscess formation, osteomyelitis, and wound dehiscence. In addition, systemic complications comprising bacteremia, hematogenous dissemination to distant tissues, and sepsis are significant^[^4^]^.

This is especially concerning in sub-Saharan Africa (SSA), where studies based in Vanuatu,^[^5^]^ Uganda,^[^6^]^ and Ethiopia^[^7^]^ have demonstrated the surgical departments are overwhelmed by subsequent SSI burden.

In a cohort study comparing pediatric patients with and without SSIs, expenses were 36% higher, with the length of admission to be extended by approximately 11 days for the SSI cohort. These are considerable increases given that only 27.3% of patients in low- to middle-income countries (LMICs) possess health insurance (See Tables 1 ).^[^2,8^]^

**Table 1:** SSI prevalence and additional costs in LMIC and HICs. Examples of surgical site infection prevalence and cost in low and high income country

Country	Prevalence	Additional cost
Kenya	7.0%	$174–$29 610 per case
United States	1.2%	$21–$34 000 per case

The financial burden related to SSIs – which includes extended hospital stays, further surgical interventions to correct the index case, and the purchase of indicated antimicrobials are of much a concern in SSA.^[^9^]^ Comparing costs associated with SSI therapeutics in LMICs to high-income countries (HICs) showed that an additional $174–$29 610 and $21–$34 000, respectively, was being spent on SSI-involved cases.^[^2^]^ This demonstrates the significant cost implication of SSI in LMICs such as SSA, taking note of the landslide difference in income across these two populations.^[^2^]^

An increased susceptibility to infections is a result of patient-related factors like that of elevated BMI and co-morbidities such as diabetes mellitus.^[^10^]^ Furthermore, HAIs are resistant to a plethora of antibiotics, thereby making them arduous to manage. Methicillin-resistant *Staphylococcus aureus* is responsible for an estimated 10% of post-operative wound infections in patients undergoing general surgery.^[^1^]^ In addition, a study in Chad found multi-resistant bacteria, like that of *Escherichia coli* and *Klebsiella pneumoniae*, to be the main causative organisms attributed to SSI.^[^11^]^ Consequently, the aim of this editorial is to thoroughly discuss the epidemiology, risk factors, and preventative measures that must be implemented to mitigate SSI in SSA.

## Epidemiology

SSI constitutes a major proportion of HAIs in LMICs. One in ten post-operative patients are likely to acquire such infections, hosting a risk of substantial morbidity, mortality, and mass economic impact.^[^12-14^]^ According to the World Health Organization (WHO) estimates, the burden of HAIs is 2 to 3 times higher in LMICs than HICs. The WHO has further reported that 15 in 100 hospitalized patients at any given time in developing countries is likely to acquire a HAI.^[^15,16^]^

With an overall reported prevalence of 14.8%, a systematic review and meta-analysis in 2020 revealed that the greatest incidence of SSI has been observed in SSA, especially the domiciles of concern to the WHO Africa. Antimicrobial resistant organisms to the most widely employed antibiotics are the primary cause of over 50% of SSIs.^[^17^]^

Acquisition of SSI has been correlated with a prolonged hospital admission ranging from 7 to 10 days.^[^18^]^ In addition, data indicate that a reduced incidence of SSI is associated with minimally invasive surgical techniques.^[^17,19,20^]^ It has been demonstrated that SSI places a clinical and financial strain on patients recovering from surgery, carers, and healthcare systems. A declination in quality of life, extended periods hospital admissions, higher incidence of postoperative pain, increased medical expenses, morbidity, and mortality are consequential.^[^13,15,19^]^

According to research conducted in several areas of Northeast Ethiopia, 12.13% of surgical patients presented with bacterial SSI overall.^[^21^]^ In contrast, the total SSI incidence in Rwanda was 10.9%^[^22^]^. Tanzania has an extremely high prevalence of stroke, up to 41.9%^[^23^]^

In contrast, Europe exhibited an incidence of 2.5% which is significantly lower than that compared with SSI rates in SSA^[^24^]^. This highlights the gap in the healthcare system between developing and developed countries, encouraging toward finding dynamic solutions.

Tables 2 and 3 demonstrate the prevalence of SSI in different countries. It is clear that there is a gap between sub-Saharan countries and developed countries. ^[^21,22,45-48^]^

**Table 2:** Prevalence of SSI in LMICs

Low-income countries	
Country	Prevalence
Rwanda	10.9%
Kenya	7.0%
Ethiopia	12.3%

**Table 3:** Prevalence of SSI in HIC

High-income countries	
Country	Prevalence
Germany	4.9%
France	0.83%
United States	1.2%

## Risk factors

According to several studies that have been conducted in SSA, the predisposing factors for SSI may be classified according to the patient status, the procedure, the hospital environment, and the practices of the healthcare team.^[^25^]^ Risk factors that are consistently identified include co-morbidities, advancing age, complexity of surgical intervention, hospital patient volume, elevated BMI, and antimicrobial resistance.^[^12,16,21,26^]^

One common etiology of heightened SSI rates is that of improper antimicrobial administration, precipitating to the development of multidrug-resistant bacteria, making preoperative antibiotic prophylaxis insufficient which in turn increases the risk for SSI.^[^21^]^

Patients with longer hospitalization are at an increased risk for SSI. Furthermore, surgeries installed with proper aseptic techniques displayed a lower SSI rate (6.06%) compared to those with insufficient sterilization measures (32.43%).^[^27^]^ In addition, diabetes mellitus had been significantly associated with increased risk of SSI.^[^26,28^]^

Most emergency surgical procedures are associated with a reduction in wound healing processes compared to their elective counterparts. This stipulates why patients who undergo emergency surgery demonstrate a greater risk of SSI acquisition.^[^29,30^]^ One important concern is ensuring adherence to the gold-standard sterilization protocols.A study showed that proper sterilization techniques are being followed in as low as a rate of 26.3% in SSA. Furthermore, as little as 47.3% of individuals complied with temperature monitoring, among many other variables.^[^2^]^

Some studies conducted in SSA have concluded that males are at a higher risk of suffering from SSI compared to females. This has been related to multiple predisposing factors observed to be more prevalent among males, most commonly cigarette smoking and being human immunodeficiency virus positive.^[^23^]^ On the contrary, females were found to be more susceptible to SSI in cases of premature rupture of the membrane pre-caesarean section.^[^31^]^

Political and economic instability impede the ability to control SSI in SSA countries. Conflicts, low healthcare budgets and socio-economic disparity found to significantly increase the burden of surgical site infections.^[^32,33^]^

Table 4 summarizes the common risk factors:^[^14,34-37^]^

**Table 4:** Risk factors of SSI in Kenya and Ethiopia. Summary of risk factors for surgical site infections in Kenya and Ethiopia reflecting the challenges in the sub-Saharan countries

Country	Common risk factors
Kenya	Long procedure duration Non-compliance to infection control practices Use of non-sterile equipments
Ethiopia	Insufficient perioperative antibiotic prophylaxis Crowded hospitals with limited resources Improper and deficient wound care

### Challenges in sub-Saharan Africa

Fully aware of the challenges healthcare systems in SSA encounter, the African Epidemiological Association Meeting in Mozambique organized a study with participants selected from 11 African nations to assess and offer potential participant-generated solutions pertaining to these issues. Inadequate human resources (34.29%), financial constraints (30%), and ineffective management of medical facilities (8.45%) were among the top three factors addressed. Furthermore, meeting participants proffered two solutions: the first attributable to a medical education program that enhanced healthcare personnel’s comprehension and commitment to SSI preventative measures (29.69%), and the second being that of a higher healthcare budget (20.31%).^[^38^]^Healthcare practitioners exhibiting inadequate training amongst ignorance of the most modern infection prevention methods were made prominent issues. Ongoing awareness and educational initiatives that bolster the skills and knowledge of healthcare professionals may ensure the best possible adherence to preventative measures.^[^39^]^

In another study, SSA was described as a region that suffered 27% of the world’s total burden of disease, yet seriously falling behind in health information technology. Attempts made to address said issue have mostly generated inconsistent or incomplete data.

### Implementing effective measures and proposing solutions to reduce surgical site infections (SSIs)

Preoperative patient preparation, proper hand hygiene, and sterilization of surgical instrumentation are important steps that must be adhered to perioperatively, ensuring SSI prevention.^[^40^]^ A multicenter study performed by Allegranzi *et al* in 2018 demonstrated a surgical unit-based safety program simultaneously conducted in hospitals across Kenya, Uganda, Zambia, and Zimbabwe between July 2013 and December 2015 which shows significant improvement when follow-up instructions were implemented.^[^41^]^

Preventive guidelines for SSI stipulate the correct administration of chlorhexidine-alcohol skin preparation, pressurized wound irrigation, Triclosan-coated sutures, and negative pressure wound therapy.^[^42^]^ In addition, strategies encompassing the avoidance of razors for hair removal, the maintenance of perioperative normothermia, and glycemic control were effective in reducing SSIs.^[^43^]^

Another critical step that must be considered is the selection of antibiotics for the prevention of SSIs^[^44^]^. A system of follow-up is crucial to guarantee that antibiotic prophylaxis is customized to the specific indicated surgery and the needs of the patient populace.^[^44^]^ This would guarantee higher compliance rates, from 12.8% to 39.1% in the surgical units, lower the number of SSIs, and lower the likelihood of antibiotic resistance.^[^41^]^Frequent and thorough follow-up consultations allow medical professionals to recognize and diagnose infection, allowing for appropriate action to be installed.^[^45^]^

Regarding the data surveillance from the field, Odekunle *et al* in 2019 advocated for the implementation of Electronic Health Records as a valid solution to improving patient care and accurate data collation.^[^46^]^

## Conclusion

One onerous public health concern in the Sub-Saharan countries that of surgical site infections. The reasons encompass a multitude of factors, uncontrolled comorbidities, antimicrobial resistance, urgent surgical procedure, inadequate funding, an underdeveloped healthcare system, noncompliance with national guidelines and regulations, and insufficient data collation and surveillance. Following the evidence-based infection control recommendations and monitoring antimicrobial use are essential. Moreover, pursuing external funding from global organizations might be taken into account as a solution to scarcity of resources.

## Data Availability

None.
